# Neural correlates of bradykinesia in Parkinson’s disease: a kinematic and functional MRI study

**DOI:** 10.1038/s41531-024-00783-2

**Published:** 2024-09-06

**Authors:** Elisabetta Sarasso, Andrea Gardoni, Lucia Zenere, Daniele Emedoli, Roberta Balestrino, Andrea Grassi, Silvia Basaia, Chiara Tripodi, Elisa Canu, Massimo Malcangi, Elisa Pelosin, Maria Antonietta Volontè, Davide Corbetta, Massimo Filippi, Federica Agosta

**Affiliations:** 1 Neuroimaging Research Unit, Division of Neuroscience, IRCCS San Raffaele Scientific Institute, Milan, Italy; 2 Vita-Salute San Raffaele University, Milan, Italy; 3 Department of Neuroscience, Rehabilitation, Ophthalmology, Genetics and Maternal Child Health, University of Genoa, Genoa, Italy; 4 Department of Rehabilitation and Functional Recovery, IRCCS San Raffaele Scientific Institute, Milan, Italy; 5 Neurology Unit, IRCCS San Raffaele Scientific Institute, Milan, Italy; 6 Neurorehabilitation Unit, IRCCS San Raffaele Scientific Institute, Milan, Italy; 7 IRCCS Ospedale Policlinico San Martino, Genoa, Italy; 8 Neurophysiology Service, IRCCS San Raffaele Scientific Institute, Milan, Italy

**Keywords:** Parkinson's disease, Parkinson's disease

## Abstract

Bradykinesia is defined as a “*complex*” of motor alterations including decreased movement amplitude and/or speed and tendency to reduce them with movement repetition (sequence effect). This study aimed at investigating the neural and kinematic correlates of bradykinesia during hand-tapping in people with Parkinson’s disease (pwPD) relative to healthy controls. Twenty-five pwPD and 25 age- and sex-matched healthy controls underwent brain functional MRI (fMRI) during a hand-tapping task: subjects alternatively opened and closed their right hand as fully and quickly as possible. Hand-tapping kinematic parameters were objectively measured during the fMRI task using an optical fibre glove. During the fMRI task, pwPD showed reduced hand-tapping amplitude (hypokinesia) and a greater sequence effect. PwPD relative to healthy controls showed a reduced activity of fronto-parietal areas, middle cingulum/supplementary motor area (SMA), parahippocampus, pallidum/thalamus and motor cerebellar areas. Moreover, pwPD showed an increased activity of brain cognitive areas such as superior temporal gyrus, posterior cingulum, and cerebellum crus I. The decreased activity of cerebellum IV–V–VI, vermis IV–V, inferior frontal gyrus, and cingulum/SMA correlated with hypokinesia and with the sequence effect. Interestingly, a reduced activity of areas involved in motor planning and timing correlated both with hypokinesia and with the sequence effect in pwPD. This study has the major strength of collecting objective motor parameters and brain activity simultaneously, providing a unique opportunity to investigate the neural correlates of the “bradykinesia complex”.

## Introduction

Bradykinesia stands out as one of the characteristic signs of parkinsonism and serves as a diagnostic criterion for Parkinson’s disease (PD)^1,2^. Its impact on upper limb functions extends beyond mere motor impairment, leading to limitations in everyday activities such as writing, using smartphones/technological devices, engaging in hobbies, and significantly affecting critical aspects of personal independence, such as dressing and personal hygiene^3–5^. Therefore, bradykinesia emerges as a fundamental sign that profoundly influences the quality of life of people with PD (pwPD)^6^.

Recent evidence^7^ suggested that it is no longer accurate to refer solely to bradykinesia as the progressive reduction in amplitude and/or speed with the repetition of movement. Instead, a broader concept of “*bradykinesia complex*” has been proposed^7^. This new paradigm recognises the presence of multiple motor manifestations including bradykinesia, hypokinesia, akinesia, oligokinesia, sequence effect and hesitations/halts^7–9^. Such complexity necessitates further detailed examination of both clinical and neurophysiological dimensions. From a clinical perspective, the Movement Disorder Society-revised Unified Parkinson’s Disease Rating Scale (MDS-UPDRS) is widely used to assess bradykinesia, but it heavily relies on the operator’s observation and does not provide quantitative information about movement^10^. New technologies, including inertial sensors, electromagnetic sensors, and stereophotogrammetry, enable the evaluation of specific parameters such as movement amplitude, acceleration, speed, and the tendency to reduce movement amplitude or speed^10–13^. These devices might facilitate the objective identification of various manifestations of the *bradykinesia complex*.

The neural correlates of the *bradykinesia complex* are also under investigation. Several studies assessed the neurophysiological correlates of bradykinesia, using techniques such as electroencephalogram (EEG), functional near-infrared spectroscopy (fNIRS), transcranial magnetic stimulation (TMS) and magnetic resonance imaging (MRI)^8,11,12^. According to previous evidence, bradykinesia mainly depends on nigrostriatal dopaminergic depletion and on altered basal ganglia activity^8^. However, growing evidence is also suggesting the involvement of sensorimotor cortical areas and cerebellum^8,12^, which play a key role in the correct execution of repetitive and continuous movements^13,14^. The presence of a dysfunctional motor network including basal ganglia, cortical motor areas and cerebellum, and the possible altered integration of sensory information both at cortical and subcortical levels can contribute to the occurrence of different movement alterations, supporting the presence of a *bradykinesia complex*^11,15^. However, further studies are needed to disentangle this hypothesis.

Among the various techniques used to investigate neural correlates of bradykinesia, MRI certainly has several advantages. Functional MRI (fMRI) allows task-specific analysis, facilitating a direct investigation of neural correlates during hand/finger-tapping^16^. Integrating fMRI and MRI-compatible devices, such as optical fibre sensors, could offer valuable understanding into the neural mechanisms underlying the complex phenomenon of bradykinesia in pwPD. For instance, an MRI-compatible optical fibre glove would enable the collection of kinematic aspects such as amplitude and velocity of hand movements simultaneously with fMRI brain activity acquisition^17^. Previous studies have successfully utilised optical fibre gloves to quantify hand/finger-tapping parameters in pwPD and to assess hand/finger motion data during fMRI in healthy subjects^17–19^.

Our hypothesis is that by simultaneously studying bradykinesia from both kinematic and neurophysiological perspectives, we could delve deeper into the neural mechanism underlying this complex phenomenon. We expect to highlight the presence of the sequence effect, which characterises patients with PD^8^. Furthermore, considering the complexity of the bradykinesia phenomenon, we expect to observe alterations not only in the basal ganglia but in a more extended brain network including cortical and cerebellar sensorimotor and cognitive areas involved in the planning, execution, and control of the spatio-temporal parameters of movement.

The aim of this study was to investigate the neural correlates of the *bradykinesia complex* in pwPD. To achieve this goal, we employed fMRI in conjunction with an MRI-compatible optical fibre glove, enabling a simultaneous exploration of neural and kinematic correlates of bradykinesia during a hand-tapping task in pwPD.

## Results

### Participants

Twenty-five pwPD and 25 age- and sex-matched healthy subjects were recruited. The two groups were similar for sociodemographic variables and MMSE score (Table 1). PwPD had a mean disease duration of 4.06 ± 3.79 years. Mean MDS-UPDRS-III was 33.64 ± 10.66 during OFF medication state and 28.25 ± 8.08 during ON state (Table 1).

**Table 1:** Sociodemographic and clinical variables in HC and pwPD

	HC (*N* = 25)	pwPD (*N* = 25)	*p* pwPD vs HC
Age (years)	64.85 ± 6.18 (53.71; 76.75)	63.73 ± 7.22 (50.99; 79.12)	0.53
Sex (M/F)	17/8	17/8	1.00
MMSE	29.12 ± 0.88 (28; 30)	29.28 ± 1.06 (25; 30)	0.33
Disease duration (years)	–	4.06 ± 3.79 (0.5; 17)	–
LEDD (mg)	–	425.56 ± 287.48 (0; 1090)	–
H&Y ON (NA/1/2/2.5)	–	1/1/22/1	–
H&Y OFF (1/2/2.5/3)	–	1/20/3/1	–
MDS-UPDRS part II	–	9.88 ± 5.70 (1; 21)	–
MDS-UPDRS part III ON	–	28.25 ± 8.08 (13; 49)	–
MDS-UPDRS part III OFF	–	33.64 ± 10.66 (13; 53)	–
PDQ-39	–	17.54 ± 12.73 (0.52; 53.74)	–

Values are mean ± standard deviation (minimum; maximum). Categorical variables are reported as frequency. *p* values refer to Mann–Whitney test or Chi-square test for categorical variables.

*HC* healthy control, *H&Y* Hoehn and Yahr score, *LEDD* levodopa equivalent daily dose, *mg* milligrams, *MDS-UPDRS-II or III* Movement Disorder Society Unified Parkinson’s Disease Rating scale part II or part III, *M/F* male/female, *MMSE* Mini-Mental State Examination, *N* Number, *NA* not applicable (a patient not taking PD medication), *PDQ-39* Parkinson’s Disease Questionnaire-39, *pwPD* people with Parkinson’s Disease.

Statistical significance: *p* < 0.05.

### Kinematic results (optical fibre glove)

**Fig. 1:**
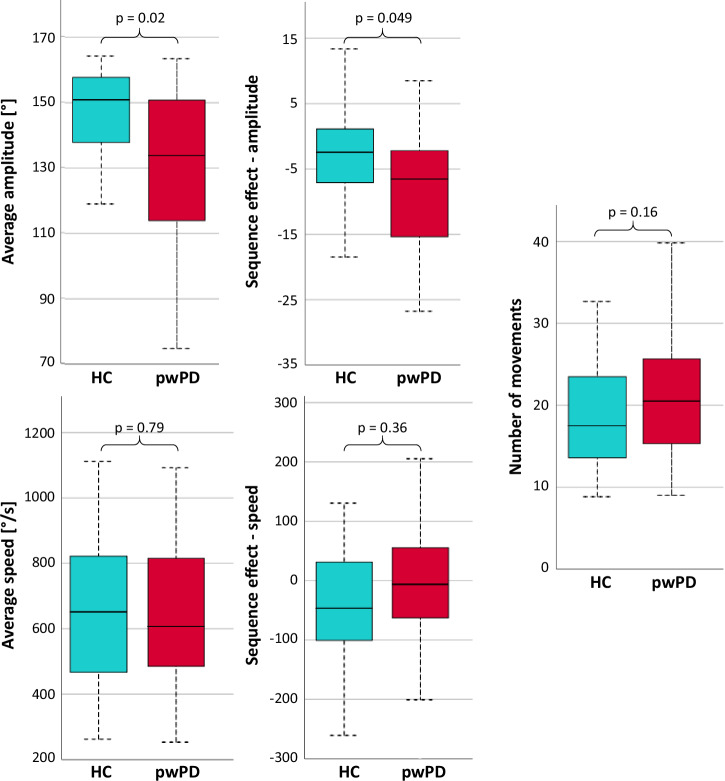
Kinematic parameters assessed with 5DT Data Glove during the fMRI hand-tapping task with the right hand in HC and pwPD. Box plot centre line refers to median; box limits refer to first and third quartiles; whiskers refer to minimum and maximum values. *p* values refer to Mann–Whitney test. Statistical significance: *p* < 0.05. HC healthy controls, pwPD people with Parkinson’s Disease, s seconds.

PwPD showed a lower average hand-tapping amplitude (hypokinesia) and a greater tendency to reduce the movement amplitude with task repetition (sequence effect) relative to healthy controls (Fig. 1 and Supplementary Table 1).

### Functional MRI results—hand-tapping task

Both healthy controls and pwPD showed activation of task-related areas such as left supplementary motor area (SMA), left primary motor cortex (M1), left fronto-parietal cortices and right cerebellum (Supplementary Fig. 1).

**Fig. 2:**
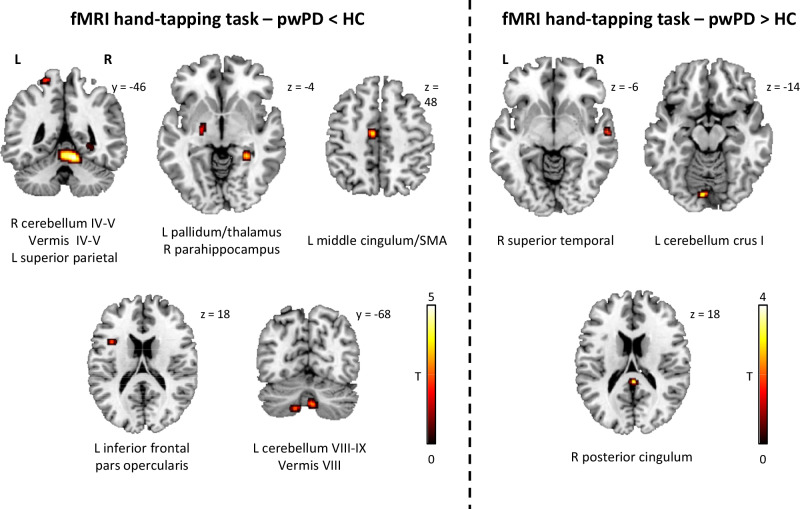
FMRI differences between pwPD and healthy controls during the right hand-tapping task. All findings are shown at *p* < 0.001 uncorrected (5000 permutations), only cluster >5 voxels are reported. Colour bars denote *T* value. Results are shown on axial, sagittal and coronal sections (*z*, *x* or *y* MNI coordinates are reported) of the Montreal Neurological Institute standard template. fMRI functional Magnetic Resonance Imaging, HC healthy controls, L left, pwPD people with Parkinson’s Disease, R right.

During hand-tapping, pwPD relative to healthy controls showed reduced activity of left middle cingulum/SMA, left cerebellum lobules VIII–IX, left pallidum/thalamus, left inferior frontal pars opercularis, left superior parietal and right parahippocampal gyri, right cerebellum lobules IV–V, cerebellar vermis IV–V and VIII (Fig. 2 and Supplementary Table 2). Moreover, pwPD relative to healthy controls showed increased activity of the right superior temporal gyrus, right posterior cingulum and left cerebellum crus I (Fig. 2 and Supplementary Table 2).

### Correlations between kinematic and functional MRI data

**Fig. 3:**
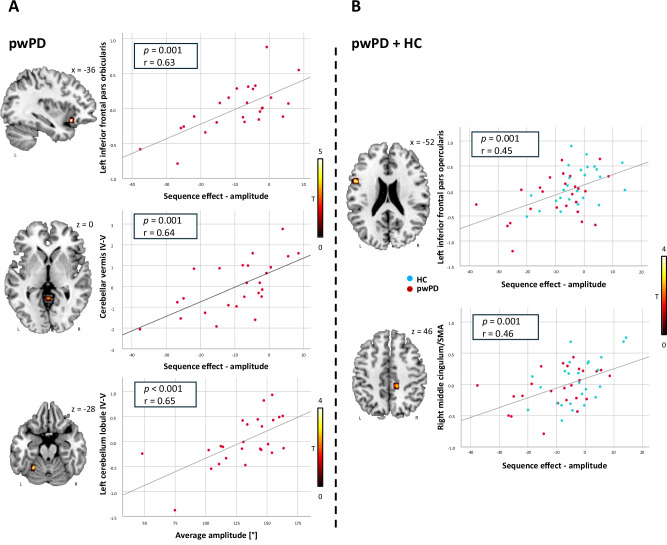
Correlation between fMRI and kinematic data obtained during the right hand-tapping task. Correlations in pwPD (**A**) and in pwPD and healthy controls together (**B**). All findings are shown at *p* < 0.001 uncorrected (5000 permutations). Colour bars denote *T* value. Results are shown on axial, sagittal and coronal sections (*z*, *x* or *y* MNI coordinates are reported) of the Montreal Neurological Institute standard template. *r* and *p* refer to Spearman’s correlation coefficient. fMRI functional Magnetic Resonance Imaging, HC healthy controls, L left, pwPD people with Parkinson’s Disease, R right.

In pwPD, a decreased activity of left cerebellum lobules IV–V correlated with a lower average hand-tapping amplitude during fMRI task, and a decreased activity of left inferior frontal gyrus pars orbicularis and cerebellar vermis IV–V correlated with a higher sequence effect on amplitude (reflecting the tendency to reduce movement amplitude with task repetition) during the fMRI task (Fig. 3 and Supplementary Table 3). In all the subjects (pwPD and healthy controls together), a decreased activity of left inferior frontal pars opercularis and right middle cingulum/SMA was associated with a greater sequence effect on amplitude during fMRI task (Fig. 3 and Supplementary Table 4).

## Discussion

The aim of the study was to investigate the neural and kinematic correlates of the bradykinesia complex assessed during a hand-tapping task in pwPD relative to healthy controls. The major strength of the study was the concurrent acquisition of hand kinematic parameters and brain activity data during fMRI hand-tapping tasks using an MRI-compatible optical fibre glove.

Compared to healthy controls, PwPD showed a global reduced hand movement amplitude and a progressive reduction in amplitude through repeated movements (sequence effect). These movement alterations are consistent with the “*bradykinesia complex*” that characterises pwPD and manifests with features such as decreased movement speed and/or amplitude, a tendency to reduce velocity and/or amplitude with repetition, and altered movement timing^7^.

Previous studies have examined bradykinesia in pwPD, highlighting the importance of a quantitative and objective assessment of movement to address the limitations associated with the lack of sensitivity and inter-/intra-rater reliability observed in commonly used clinical scales^20–24^. Currently, the majority of studies use inertial sensors to assess spatio-temporal parameters of movement as they are validated and relatively low-cost instruments^25^. However, these devices are incompatible with MRI environments and preclude real-time assessment of neural correlates associated with bradykinesia.

To obtain hand kinematic data during MRI acquisition for an ecological analysis of neural correlates associated with repeated hand movements, we used an MRI-compatible optical fibre glove (5DT Data Glove) that has been previously used in other MRI studies^26,27^. Importantly, to our knowledge, no study validated the 5DT Data glove, therefore, we first tested the reliability of the glove before its use in this study, and we found a strong correlation (*r* > 0.70, *p* < 0.001) between the motion parameters obtained with the glove and using a gold-standard motion analysis system.

Our findings regarding hand kinematics align with those of a prior study that evaluated the impact of movement repetition of the hand (finger-tapping, hand-tapping, and forearm prono-supination) using motion sensors in pwPD during OFF medication^22^. This study primarily demonstrated deficits in amplitude as opposed to alterations in speed or rhythm^22^.

During the fMRI hand-tapping task, pwPD relative to healthy controls showed a reduced activity of basal ganglia, cerebellar motor areas, middle cingulum/SMA, superior parietal and inferior frontal gyri; and an increased activity of superior temporal gyrus, posterior cingulum and cerebellum crus I. Such fMRI changes, with a mixed pattern of hypo- and hyper-activations, could be related to the coexistence of basal ganglia failure typical of pwPD^28–30^ together with the attempt to compensate through the increased activity of cognitive areas such as posterior cingulum, superior temporal gyrus and cerebellum crus I, reflecting the need of additional cognitive control to perform a simple motor task in pwPD^31–33^. Moreover, during the hand-tapping task, pwPD showed not only a reduced activity of basal ganglia but also of frontal, parietal and cerebellar motor areas suggesting a functional disconnection of the cerebello-thalamo-cortical circuit^34,35^. As already shown in previous studies^36–38^, we found a reduced activity of the SMA that is involved in motor planning, execution, and control as well as in timing and spatial processing^38,39^. Previous evidence suggested that SMA hypoactivation in pwPD is particularly evident when the task requires monitoring and paying attention to the movement^40^. Our fMRI task, despite its simplicity, required control and attention on movement as we explicitly instructed the subject to perform the hand-tapping as ample and as fast as possible. Interestingly, the reduced activity of the SMA correlated with a worse sequence effect on amplitude in pwPD, supporting the role of this area in controlling spatio-temporal parameters and planning of repetitive movements.

Moreover, we found a reduced activity of the superior parietal gyrus in pwPD relative to healthy controls. The superior parietal lobe mediates the kinaesthetic sensation, being involved in the control of the hand position during the task, and consequently playing a role in adjusting movement parameters during internally-generated movements^41,42^. The hand-tapping task we proposed required spatial control abilities and integrity of kinaesthetic sensibility (during fMRI task, subjects wore a headset that provided commands and precluded the vision of their upper limb). The reduced activity of the parietal cortex in our patients might have contributed to the difficulty in maintaining an adequate movement amplitude during the hand-tapping task^37^.

PwPD also showed a reduced activity of the inferior frontal gyrus that plays a role in motor timing and inhibition^43–45^ and is a primary component of the mirror system, important in learning and recognising actions and intentions^46^. Interestingly, we found a correlation between a reduced activity of the inferior frontal gyrus and a worse sequence effect on amplitude in both pwPD and healthy controls, suggesting that extra-motor areas may play a fundamental role in controlling and maintaining movement parameters throughout repetitions. Accordingly, a previous study found a correlation between a reduced recruitment of inferior frontal gyrus and worse freezing episodes^47^. PwPD also showed reduced activity of motor cerebellar areas that are involved in movement coordination, temporal and spatial representation of movements and predictive motion control^48^. A reduced activity of cerebellar motor areas in our pwPD patients correlated with a worse bradykinesia in terms of reduced hand-tapping amplitude and greater sequence effect on amplitude. This highlights the role of cerebellum in motor control in pwPD supporting its involvement in the pathophysiology of bradykinesia as previously suggested^8^.

Previous studies have endeavoured to elucidate the neural substrates of bradykinesia with various methodologies. Some utilised TMS in conjunction with stereophotogrammetric data^20^, while others employed apparatuses (joystick)^49,50^ or drawing tasks^23^ to obtain movement parameters during fMRI acquisitions. Although these investigations offer intriguing insights into the potential involvement of basal ganglia, cerebellum and other brain areas in bradykinesia, the absence of simultaneous collection of kinematic and brain activity data during a simple hand or finger-tapping task—an ideal approach for directly studying bradykinesia—poses a limitation in interpreting findings as merely related to bradykinesia^51^. Conversely, in our study, we evaluated both brain activity and kinematic parameters concurrently, yielding promising results that enhance our understanding of the neural correlates underlying bradykinesia.

This study is not without limitations. Despite the limited study sample size, it is imperative to acknowledge the challenges of recruiting pwPD who are able to perform fMRI trials. Notably, the pwPD participants in our study represent a population with a relatively short disease duration (mean 4 years), which may have favoured the detection of a sequence effect that is characteristic of earlier phases of the disease, before severe hypokinesia emerges, as suggested in prior research^52^. On the other hand, kinematic parameters and fMRI data might be particularly interesting in the earlier stages of PD, considering that the sequence effect is regarded as a clinical clue for distinguishing bradykinesia in PD from that seen in atypical parkinsonism^8^. In line with that, the short duration of disease is not a limitation, as milder kinematic alterations are more difficult to be recognised. It would be even more significant to perform the same analysis in de novo PD, at initial stage of hemiparkinsonism. Future studies should also perform kinematic analysis in both the ON and OFF phases to elucidate the dopaminergic mechanisms in the bradykinesia complex, particularly the sequence effect.

Furthermore, fMRI results should be interpreted carefully as we did not obtain significant findings with a family-wise error correction. To improve robustness of results, we used a permutation-based approach that has the main advantages to overcome the massive number of multiple comparisons, reduce the great effort in testing the normality in a voxel-wise analysis, and control for false positive rate.

In conclusion, our findings suggest a correlation between the *bradykinesia complex* and the hypoactivation of brain areas strongly involved in motor planning and monitoring such as SMA, motor cerebellum and inferior frontal gyrus in pwPD. This supports the idea of bradykinesia as a network dysfunction^8^. A clearer knowledge of this phenomenon could result in improved management of pwPD, and other patients with bradykinesia, both in clinical and research settings.

## Methods

### Subjects and study design

This study is part of an ongoing randomised controlled trial on the effects of physiotherapy on the upper limb in pwPD (ClinicalTrials.gov ID: NCT04876352). Right-handed outpatients with idiopathic PD^53^ were recruited at the Movement Disorder Unit, Unity of Neurology, IRCCS Ospedale San Raffaele, Milan, Italy, according to the following inclusion criteria: Hoenh & Yahr (H&Y) ≤ 3 while on medication; age ≤ 85 years; right-side involvement according to the H&Y and MDS-UPDRS-III; handwriting difficulty (MDS-UPDRS II.7 ≥ 1). Age- and sex-matched right-handed healthy controls were recruited by word of mouth among nonconsanguineous relatives and institute personnel. Exclusion criteria for both patients and healthy controls were: Mini-Mental State Examination (MMSE) < 24 in pwPD and <28 in healthy controls; visual impairments that interfere with use of screens; upper limb deficits impeding handwriting; history of (other) systemic, neurologic, psychiatric diseases, head injury or brain damage at routine MRI, including lacunae and extensive cerebrovascular disorders; history of alcohol and/or psychotropic drug abuse; denied oral and written informed consent to study participation.

PwPD and healthy controls underwent neuropsychological screening using MMSE and brain MRI scans. PwPD also underwent a neurological evaluation. An experienced neurologist performed the neurological evaluation including H&Y, MDS-UPDRS part II, and part III both during ON and OFF medication state (at least 12 h after the regular evening dopaminergic therapy administration, except for dopamine agonists that required 24-h discontinuation).

Local ethical standards committee on human experimentation (Ethical Committee IRCCS San Raffaele Scientific Institute Milan, Italy) approved the study protocol (No. 68/int/2019) and all subjects provided written informed consent prior to study participation.

### MRI protocol

Using a 3.0 Tesla Philips Intera scanner (*Ingenia CX, Philips Medical System, Best, The Netherlands)*, brain MRI scans were obtained during OFF time (at least 12 h after the regular evening dopaminergic therapy administration, except for dopamine agonists that required 24-h discontinuation), to mitigate the pharmacological effects on neural activity. Both functional and structural MRI sequences were acquired. The following structural brain MRI sequences were acquired to exclude subjects with eventual structural brain alterations and/or excessive vascular lesions: (i) 3D T2-weighted: TR = 2500 ms, TE = 330 ms, flip angle = 90°, 192 contiguous sagittal sections, thickness = 1 mm, field of view (FOV) = 256 mm × 256 mm, matrix = 256 × 258, voxel reconstruction = 0.9 mm × 0.9 mm × 1 mm; (ii) 3DT1-weighted: TR = 7.1 ms, TE = 3.2 ms, flip angle = 9°, 204 contiguous sagittal sections, thickness = 1 mm, FOV = 256 mm × 240 mm, matrix = 256 × 240, voxel reconstruction = 1 mm × 1 mm × 1 mm; (iii) 3D Flair: TR = 4800 ms; TE = 269 ms; flip angle = 40°; 192 contiguous sagittal sections; thickness = 1.5 mm; FOV = 256 × 256 mm; matrix = 256 × 256, voxel reconstruction 1 × 1 × 1.

### Functional MRI study

FMRI was obtained using a T2* weighted echo planar imaging sequence with the following parameters: echo time (TE) = 35 ms, repetition time (TR) = 1572 ms, flip angle = 70°, field of view (FOV) = 240 × 240 mm, matrix = 96 × 94, 48 contiguous axial sections, thickness = 3 mm, voxel reconstruction 2.5 × 2.5 × 3 mm. During the fMRI hand-tapping task, subjects were asked to open and close the right hand as fully and quickly as possible for about 10 s (according to the instruction of MDS-UPDRS item 3.5) while wearing an MRI-compatible optical fibre glove. A block design (ABAB) was used during the task: activation periods (A) corresponded to the execution of the hand-tapping (six hand-tapping periods lasting about 10 s each), while rest periods (B) represented resting periods without movements (six resting periods lasting about 10 s each). Subjects wore also an MRI-compatible headset which projected the commands to start and stop the task and a fixed cross during both the hand-tapping movements and the resting period. Sandbags were used to position participants’ hands and prevent unwanted movements. Before entering the scanner, patients familiarised with the hand movement required during the task.

### Optical fibre glove

We used the optical fibre 5DT Data Glove 14 Ultra (Fifth Dimension Technologies Inc., Orlando, USA), a one-sized hand motion capture device that measures finger flexion and abduction. Finger flexions are measured at metacarpal and proximal interphalangeal joints using 14 optical fibre sensors with a resolution of 12-bit A/D (typical range 10 bits) embedded in a stretch lycra glove that allows the proper fit on the hand. Fibre loops are connected to a LED, and the glove measures finger flexion indirectly based on the intensity of the light returned to a phototransistor. The system interfaces with the computer via a full-speed USB cable. An ad-hoc customised software was developed in order to record signals from the glove. In order to assess the reliability of the 5DT Data Glove to detect and quantify movement parameters, we conducted a validation study using an optoelectronic system as gold standard in a group of healthy young volunteers.

The study was conducted using a stereophotogrammetric system SMART DX 7000 (BTS Bioengineering, Garbagnate Milanese, Italy) equipped with six optoelectronic cameras gold standard. Participants were positioned at the centre of the stereophotogrammetric system acquisition volume with their arm along the chest and the elbow flexed at 135°. Participants wore the 5DT Data Glove and three reflective markers placed on their hand, over the glove. Two markers were laced on the second metacarpal bone (on the base and on the head) and one on the second proximal phalanx of the second finger. Starting from a fully open hand position, subjects were asked to alternatively open and close (full flexion of metacarpophalangeal and proximal interphalangeal joints with distal interphalangeal joint in neutral position) their hand performing ample movements (hand-tapping). The hand-tapping was performed at two different speeds, guided by auditory cues at the frequency of 1 and 3 Hz, representing comfortable and fast movement speed, respectively. Subjects were instructed to open or close their hand at each auditory stimulus performing four full hand tap movements. A customised software provided: (i) visual instruction about when to start/stop the hand-tapping on a monitor in front of the subject and (ii) auditory signals guiding movement speed.

There was a 6-s rest period between 1 and 3 Hz acquisitions. Each condition was performed once. Before acquisition the subjects were trained to correctly follow the auditory cues.

We carried out an a-priori power analysis^54^. Assuming a strong correlation (|R| = 0.7)^55,56^ between the data from the two motion capture systems in each subject and a null hypothesis of |R| = 0.5, 98 values (frames) would be needed for 80% power to detect a significant correlation (*a* = 0.05) between the data from the two systems. For each subject, correlation between data from the glove and the optoelectronic system was assessed using Spearman’s rank correlation coefficient (0.3 ≤ |R| < 0.5 low correlation; 0.5 ≤ |R| < 0.7 moderate correlation; 0.7 ≤ |R| < 0.9 strong correlation; 0.9 ≤ |R| < 1 very strong correlation) for each condition (1 and 3 Hz).

Statistical significance was set at *p* < 0.05. Mean values of Spearman’s correlation coefficient among subjects were then calculated for each condition showing the overall correlation between the two signals. Statistical analyses were conducted using IBM SPSS software (version 25.0). We observed a strong correlation between the hand-tapping motion parameters obtained with the gold-standard optolectronic system and with the optical fibre glove (see Supplementary Material for the results), we then used the optical fibre glove to acquire hand-tapping motion parameters in the fMRI setting.

### Hand-tapping kinematic parameters’ analysis

Hand-tapping kinematic parameters as registered with the optical fibre glove were analysed using a customised software. Subjects performed six hand-tapping periods. The software automatically detected peaks of amplitude and counted the number of gestures based on the alternation of high (open hand) and low (closed hand) peaks (only movements >20° of excursion of metacarpophalangeal joints were considered). An operator visually examined on a computer screen the amplitude and speed data profile for each hand-tapping period to ensure that the automatised process worked correctly. For each of the six hand-tapping periods, the software extracted the number of hand-tapping movements, mean movement amplitude and speed of all the fingers, and the tendency to modify movement speed and/or amplitude with task repetition (sequence effect). The sequence effect was calculated as the slope of the line connecting the peaks of amplitude and speed values, respectively. Parameters from the six hand-tapping periods were mediated to conduct the analyses.

### Functional MRI Analysis

Prior to statistical analysis, all images were realigned to the first one to correct for subject motion (all study participants showed maximal head movements lower than 3 mm in each direction), slice-timing corrected, coregistered to subject’s 3DT1, spatially normalised into the standard MNI (Montreal Neurological Institute) space, and smoothed applying a 8-mm 3-D Gaussian filter. The signal variations of the BOLD effect associated with the hand-tapping task (considering movement parameters as confounds) were evaluated voxel by voxel using the General linear model (GLM) and Gaussian field theory. Specific effects were tested applying appropriate linear contrasts. Significant hemodynamic changes for each contrast were evaluated using a nonparametric permutation-based approach, that is Statistical nonParametric Mapping (SnPM), a toolbox for SPM (http://www.fil.ion.ucl.ac.uk/spm; Wellcome Trust Center for Neuroimaging, London, UK)^57^.

### Statistical analysis

Sociodemographic, clinical and kinematic data were compared between groups using Chi-square test for categorical variables and Mann–Whitney test for continuous variables using IMB SPSS Software (Version 25.0). The significance level was set at a *p* < 0.05 (two-sided).

Regarding fMRI analysis, the one-sample *t*-test was used to evaluate significant mean brain activations of each group during the hand-tapping task; differences between groups (healthy controls vs pwPD) were assessed using a two-sample *t*-test. Multiple linear regression models were used to assess the correlation between fMRI activity and kinematic data detected by the 5DT Data Glove during hand-tapping tasks. For all permutation-based contrasts, nonparametric testing was performed with 5000 random permutations. All findings are shown at *p* < 0.001 uncorrected, and only clusters >5 voxels were considered.

## Supplementary information

Supplemental material

## Data Availability

The dataset used and analysed during the current study is available from the corresponding author upon request to qualified researchers (i.e., affiliated to a university or research institution/hospital).
