# Supplementary material for: Neural correlates of bradykinesia in Parkinson’s disease: a kinematic and functional MRI study
Source: NPJ Parkinsons Dis. 2024 Sep 6;10:167. doi: 10.1038/s41531-024-00783-2 (PMC11379907; doi:10.1038/s41531-024-00783-2)
Supplement: Supplementary file 1 — Supplemental material [file 41531_2024_783_MOESM1_ESM.docx]

**SUPPLEMENTARY MATERIALS**

**RESULTS**

**Supplementary table 1** - Kinematic parameters assessed with 5DT Data Glove during the fMRI hand-tapping task with the right hand in healthy controls and pwPD.

|  | **HC**  **(N=25)** | **pwPD**  **(N=25)** | **p**  **pwPD vs HC** |
| --- | --- | --- | --- |
| **Number of movements** | 18.52 ± 6.74  (8.83; 32.67) | 21.62 ± 7.70  (9.00; 39.83) | 0.16 |
| **Average amplitude [°]** | 147.28 ± 13.27  (119.02; 164.22) | 130.56 ± 27.94  (48.12; 163.44) | **0.02** |
| **Sequence effect – amplitude** | -2.83 ± 8.37  (-19.98; 14.24) | -9.45 ± 11.32  (-37.65; 8.52) | **0.049** |
| **Average speed [°/s]** | 665.26 ± 228.11  (262.78; 1112.02) | 655.66 ± 234.15  (253.81; 1092.39) | 0.79 |
| **Sequence effect - speed** | -31.73 ± 111.88  (-260.57; 275.31) | -9.85 ± 117.94  (-292.05; 254.51) | 0.36 |

*Values are mean ± standard deviation (minimum; maximum). p values refer to Mann-Whitney test. Statistical significance: p < 0.05.* ***Abbreviations****: HC = healthy controls; pwPD = people with Parkinson’s Disease; s = seconds.*

**Supplementary table 2** – FMRI pattern of activation in healthy controls relative to pwPD during hand-tapping task performed with the right hand.

| **Hand-tapping task** | | | | | | |
| --- | --- | --- | --- | --- | --- | --- |
| **pwPD < HC** | | | | | | |
| **kE** | **T** | **mni X** | **mni Y** | **mni Z** | **BA** | **Area** |
| 337 | 5.07 | 10 | -46 | -16 | - | Right cerebellum lobules IV-V |
|  | 4.35 | -2 | -46 | -12 | - | Cerebellar vermis IV-V |
| 33 | 4.39 | 28 | -42 | -4 | 37 | Right parahippocampal gyrus |
| 27 | 4.20 | -8 | -12 | 48 | - | Left middle cingulum/SMA |
| 11 | 4.01 | -20 | -48 | 70 | 5 | Left superior parietal gyrus |
| 32 | 3.82 | -16 | -64 | -46 | - | Left cerebellum lobules VIII-IX |
| 21 | 3.74 | 4 | -68 | -42 | - | Cerebellar vermis VIII |
| 8 | 3.60 | -22 | -14 | -4 | - | Left pallidum/thalamus |
| 12 | 3.46 | -36 | 10 | 20 | 48 | Left inferior frontal pars opercularis |
| **pwPD > HC** | | | | | | |
| 6 | 3.62 | 60 | -12 | -6 | 22 | Right superior temporal gyrus |
| 17 | 3.60 | -6 | -84 | -14 | 17 | Left cerebellum crus I |
| 38 | 3.55 | 2 | -38 | 18 | 26 | Right posterior cingulum |

*X, y and z coordinates refer to the Montreal Neurological Institute (MNI) space.* *Table reports results of the two-sample t-test performed in SnPM with 5000 random permutations (p < 0.001 uncorrected). Only clusters greater than 5 voxels are reported.*  ***Abbreviations:*** *BA = Brodmann Area; HC = healthy controls; kE = cluster extension; mni = Montreal Neurological Institute; pwPD = people with Parkinson’s Disease.*

**Supplementary table 3** - Correlations between fMRI and kinematic data obtained during hand-tapping task in pwPD.

| **pwPD** | | | | | | | | | |
| --- | --- | --- | --- | --- | --- | --- | --- | --- | --- |
| **Hand-tapping task – Average amplitude (°)** | | | | | | | | | |
| **p** | **r** | **+/-** | **kE** | **T** | **mni X** | **mni Y** | **mni Z** | **BA** | **Area** |
| <0.001 | 0.65 | + | 11 | 3.81 | -28 | -52 | -20 | - | Left cerebellum lobule IV-V |
| **Hand-tapping task – Sequence effect - amplitude** | | | | | | | | | |
| 0.001 | 0.63 | + | 14 | 4.54 | -36 | 22 | -12 | 48 | Left inferior frontal gyrus pars orbicularis |
| 0.001 | 0.64 | + | 8 | 3.94 | 0 | -50 | 0 | - | Cerebellar vermis IV-V |

*p and r refer to Spearman’s correlation. Positive correlation (+) means that both fMRI brain activity and clinical value decrease or increase. X, y and z coordinates refer to the Montreal Neurological Institute (MNI) space.* *Table reports results of the multiple linear regression models performed in SnPM with 5000 random permutations (p < 0.001 uncorrected) . Only clusters greater than 5 voxels are reported.* ***Abbreviations:*** *+/- = positive/negative correlation; BA = Brodmann Area; kE = cluster extension; mni = Montreal Neurological Institute; pwPD = people with Parkinson’s Disease.*

**Supplementary table 4** – Correlations between fMRI and kinematic data during hand-tapping task in pwPD and HC.

| **pwPD + HC** | | | | | | | | | |
| --- | --- | --- | --- | --- | --- | --- | --- | --- | --- |
| **Hand-tapping task – Sequence effect - amplitude** | | | | | | | | | |
| **p** | **r** | **+/-** | **kE** | **T** | **mni X** | **mni Y** | **mni Z** | **BA** | **Area** |
| 0.001 | 0.45 | + | 24 | 3.94 | -52 | 8 | 22 | 44 | Left inferior frontal pars opercularis |
| 0.001 | 0.46 | + | 14 | 3.70 | 14 | -36 | 46 | - | Right middle cingulum/SMA |

*p and r refer to the Spearman’s correlation. Positive correlation (+) means that both fMRI brain activity and clinical value decrease or increase. X, y and z coordinates refer to the Montreal Neurological Institute (MNI) space.* *Table reports results of the multiple linear regression models performed in SnPM with 5000 random permutations (p < 0.001 uncorrected). Only clusters greater than 5 voxels are reported.* ***Abbreviations:*** *+/- = positive/negative correlation; BA = Brodmann Area; HC = healthy controls; kE = cluster extension; mni = Montreal Neurological Institute; pwPD = people with Parkinson’s Disease; SMA = supplementary motor area*.

**
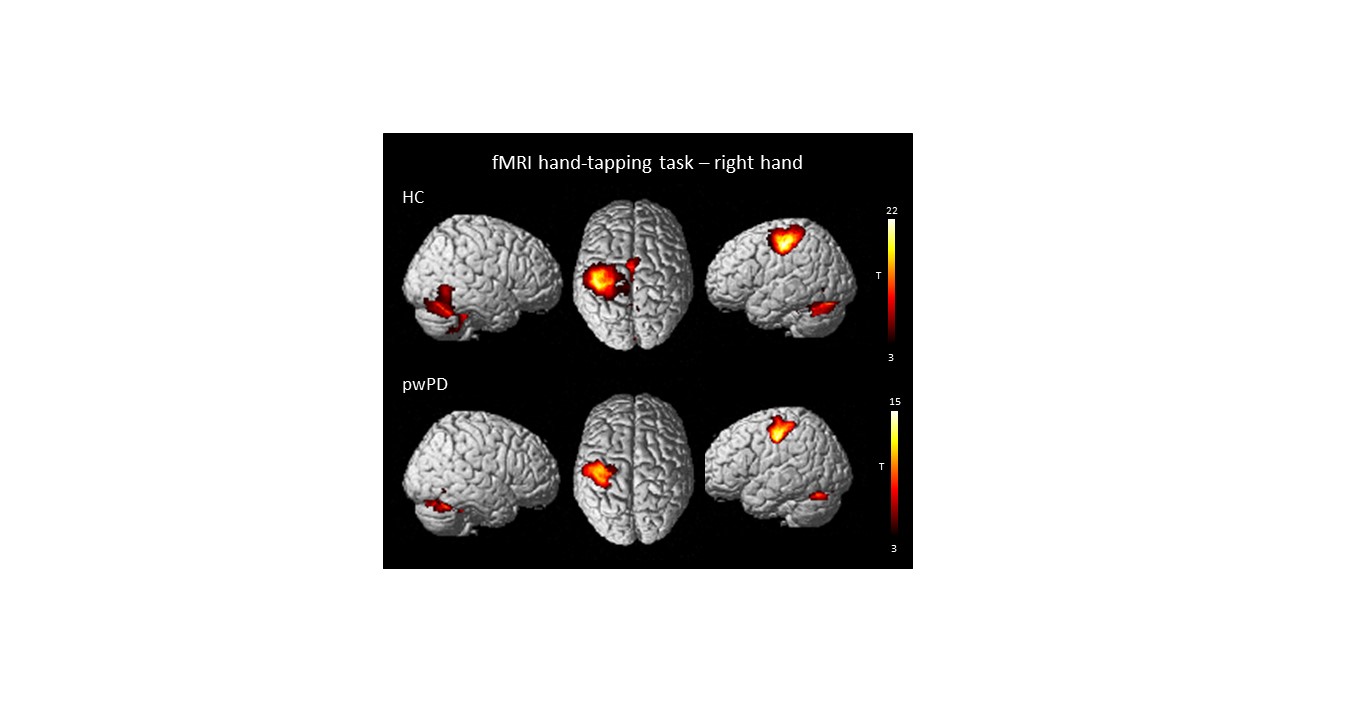
**

**Supplementary figure 1 -** FMRI patterns of activation in healthy controls and pwPD during the right hand-tapping task shown on a rendered brain. Findings are shown at p <0.001 uncorrected (5000 permutations). Color bars represent T value. **Abbreviations:** fMRI = functional Magnetic Resonance Imaging; HC = healthy controls; pwPD = people with Parkinson’s Disease.

***Results of the 5DT Data Glove validation***

Fifteen volunteers (8 males, 53.3%) with a mean age of 22.8 ± 1.2 years, a mean height of 179 ± 10 cm and a mean BMI 22.07 ± 1.53 kg/m2, were enrolled. Participants hand size ranged from 7.60 to 9.10 cm in width and from 17.30 to 20.50 cm in height, with a span from 19.80 to 24.70 cm (Supplementary table 5).

**Supplementary table 5 –** Demographic and biometric characteristics of subjects included in the validation study.

| **Healthy controls (N = 15)** | | |
| --- | --- | --- |
|  | **Mean ± standard deviation**  **(min; max)** | **Median**  **(1^st^-3^rd^ quartiles)** |
| **Age [years]** | 22.77 ± 1.17 (21.56; 25.77) | 22.39 (21.99 – 1.18) |
| **Sex [M/F]** | 8/7 | |
| **Height [cm]** | 179 ± 10 (160; 194) | 178 (172 – 186) |
| **Weight [kg]** | 70.67 ± 9.34 (53; 85) | 74 (63.5 – 75.5) |
| **BMI [kg/m^2^]** | 22.07 ± 1.53 (19.87; 24.54) | 22.58 (20.66 – 23.15) |
| **Hand width [cm]** | 8.24 ± 0.54 (7.60; 9.10) | 8.30 (7.75 – 8.55) |
| **Hand length [cm]** | 18.67 ± 1.10 (17.30; 20.50) | 18.40 (17.65 – 19.60) |
| **Hand span [cm]** | 22.22 ± 1.61 (19.80; 24.70) | 22.10 (21.00 – 23.40) |

***Abbreviations:*** *BMI = body mass index; cm = centimeters; kg = kilograms; M/F = male/female; min; max = minimum and maximum; N = number.*

During the four hand-tapping movements at 1Hz frequency, the motion capture systems captured at least 290 frames (mean 327); during the movements at 3Hz frequency it captured at least 100 frames (mean 109). There was a mean strong significant positive correlation between the data from the two systems both during movements at 1Hz (R = 0.79) and 3Hz (R = 0.81) (supplementary table 6). Correlation was significant in each subject (p < 0.001) and each subject in each condition showed a strong correlation (R ≥ 0.7) (Supplementary figure 2 and supplementary table 7).

**Supplementary table 6 –** Spearman’s correlation coefficient between 5DT Data Glove and stereophotogrammetric system data in the group of HC.

| **Hand-tapping 1Hz (4 movements)** | | |
| --- | --- | --- |
| **15 healthy controls** | **Mean ± standard deviation**  **(min; max)** | **Median**  **(1^st^-3^rd^ quartiles)** |
| **Number of values [frames]** | 327.67 ± 21.78 (290; 360) | 320 (315 - 345) |
| **Spearman’s correlation coefficient** | 0.79 ± 0.05 (0.69; 0.86) | 0.787 (0.76 – 0.83) |
| **Hand-tapping 3Hz (4 movements)** | | |
| **15 healthy controls** | **Mean ± standard deviation**  **(min; max)** | **Median**  **(1^st^-3^rd^ quartiles)** |
| **Number of values [frames]** | 109.67 ± 6.40 (100; 125) | 110 (105 – 112.5) |
| **Spearman’s correlation coefficient** | 0.81 ± 0.058 (0.68; 0.90) | 0.82 (0.79 – 0.86) |

*R = Spearman’s correlation coefficient: 0.3 ≤ | R| < 0.5 low correlation; 0.5 ≤ | R| < 0.7 moderate correlation; 0.7 ≤ | R| < 0.9 strong correlation; 0.9 ≤ | R| < 1 very strong correlation.* ***Abbreviations:*** *HC= healthy controls; Hz = hertz; min; max = minimum and maximum.*

**Supplementary table 7 -** Spearman’s correlation coefficients between data glove and stereophotogrammetric systems in each control subject.

|  | **Hand-tapping 1Hz** | | | | **Hand-tapping 3 Hz** | | | |
| --- | --- | --- | --- | --- | --- | --- | --- | --- |
|  | **N of values**  **(frames)** | **N**  **of movements** | **R** | **p** | **N of values**  **(frames)** | **N**  **of movements** | **R** | **p** |
| sub_01 | 315 | 4 | 0.825 | <0.001 | 100 | 4 | 0.826 | <0.001 |
| sub_02 | 350 | 4 | 0.847 | <0.001 | 110 | 4 | 0.800 | <0.001 |
| sub_03 | 315 | 4 | 0.784 | <0.001 | 110 | 4 | 0.783 | <0.001 |
| sub_04 | 315 | 4 | 0.746 | <0.001 | 105 | 4 | 0.748 | <0.001 |
| sub_05 | 340 | 4 | 0.808 | <0.001 | 125 | 4 | 0.787 | <0.001 |
| sub_06 | 360 | 4 | 0.787 | <0.001 | 110 | 4 | 0.819 | <0.001 |
| sub_07 | 340 | 4 | 0.837 | <0.001 | 110 | 4 | 0.758 | <0.001 |
| sub_08 | 320 | 4 | 0.755 | <0.001 | 110 | 4 | 0.853 | <0.001 |
| sub_09 | 360 | 4 | 0.787 | <0.001 | 115 | 4 | 0.864 | <0.001 |
| sub_10 | 315 | 4 | 0.693 | <0.001 | 110 | 4 | 0.858 | <0.001 |
| sub_11 | 290 | 4 | 0.770 | <0.001 | 105 | 4 | 0.683 | <0.001 |
| sub_12 | 320 | 4 | 0.863 | <0.001 | 105 | 4 | 0.844 | <0.001 |
| sub_13 | 300 | 4 | 0.831 | <0.001 | 100 | 4 | 0.788 | <0.001 |
| sub_14 | 355 | 4 | 0.851 | <0.001 | 115 | 4 | 0.885 | <0.001 |
| sub_15 | 320 | 4 | 0.727 | <0.001 | 115 | 4 | 0.903 | <0.001 |

*R = Spearman’s correlation coefficient: 0.3 ≤ | R| < 0.5 low correlation; 0.5 ≤ | R| < 0.7 moderate correlation; 0.7 ≤ | R| < 0.9 strong correlation; 0.9 ≤ | R| < 1 very strong correlation. P values refer to Spearman’s correlation coefficient. Statistical significance was accepted for p<0.05.* ***Abbreviations:*** *Hz = hertz; N = number; sub = subject.*


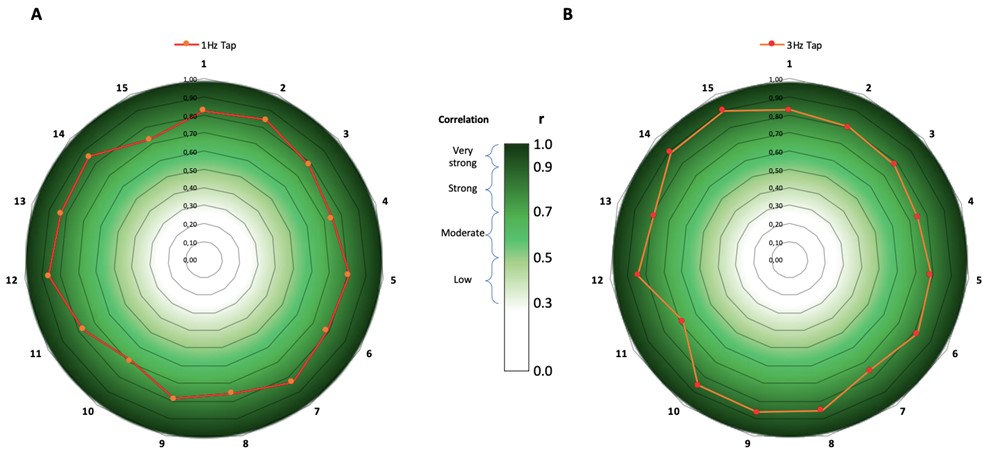


**Supplementary figure 2 –** Correlation between data obtained with the 5DT Data Glove and with the stereophotogrammetric system in young healthy volunteers. Radar plots represent the Spearman’s correlation coefficient (r) between 5DT Data Glove and the stereophotogrammetric data obtained during the hand-tapping at 1Hz (A) and 3Hz (B) in each control subject.
